# Conditional Cash Transfer to Improve TB Outcomes: Necessary but Not Sufficient: Comment on "Does Direct Benefit Transfer Improve Outcomes Among People With Tuberculosis? – A Mixed-Methods Study on the Need for a Review of the Cash Transfer Policy in India"

**DOI:** 10.34172/ijhpm.2022.7643

**Published:** 2023-01-18

**Authors:** Fernando Rubinstein, Alejandro Blumenfeld

**Affiliations:** ^1^Institute of Clinical Effectiveness and Health Policy, Buenos Aires, Argentina.; ^2^Division of Population Health, Health Services Research and Primary Care, University of Manchester, Manchester, UK.

**Keywords:** Tuberculosis, Social Support, Poverty, Health Policy

## Abstract

Tuberculosis (TB) still represents a major public health problem in many regions of the world. TB control can only be achieved through a comprehensive and inclusive response which takes into account both upstream and downstream coordinated interventions related to structural determinants such as poverty, nutrition, sanitation, housing and access to healthcare as well as timely diagnosis and support throughout the course of treatment. Several social and financial support strategies have been proposed to improve TB treatment adherence, including conditional cash transfers (CCTs). In this context, demonstrating that social protection directly improves a specific health outcome using routinely collected data, incomplete registries or surveillance reports brings about many methodological challenges. We briefly discuss this paper and some limitations, describe main findings from our own research in this area and make a call to expand social protection interventions to address structural conditions of those most affected.

 Tuberculosis (TB) still represents a major public health problem in many regions of the world, affecting almost exclusively the most socially and economically deprived groups. In the last years, the coronavirus disease 2019 (COVID-19) pandemic and the impact resulting from the financial and societal consequences associated with the containment measures in the short- and long-term, deteriorated the situation of the most vulnerable and deepened the socioeconomic determinants of TB, resulting in a significant delay in timely diagnosis and treatment. Although treatment is widely available, highly effective and provided for free in most public systems worldwide, it is long and burdensome for many patients, leading to suboptimal results due to low adherence to treatment. Adherence is a fundamental factor to achieve TB control and we need good quality evidence of the effect of policy interventions to improve its levels and overcome this important barrier to success.

 TB control can only be achieved through a comprehensive and inclusive response which takes into account both upstream and downstream coordinated interventions related to structural determinants such as poverty, nutrition, sanitation, housing and access to healthcare as well as timely diagnosis and support throughout the course of treatment.^1^ Several strategies have been proposed to improve TB treatment adherence by assisting household economy and compensating income loss using financial incentives to affected individuals and their families, such as conditional cash transfers (CCTs). Economic support measures to improve health outcomes have been gradually introduced in the 1990s decade in different contexts and health subjects such as infant mortality and maternal/perinatal health, vertical HIV transmission, mental health, sexual and reproductive health, among others.^2^ CCTs have also been studied regarding TB-specific outcomes, as there is global recognition of multiple socioeconomic barriers that negatively influence treatment access and adherence.^3^ CCTs can offer a positive incentive to complete TB treatment and hence improve health outcomes, but to date, although on the rise, there is still limited evidence on the direct effectiveness of socioeconomic support interventions in TB.

 Dave and Rupani conducted a mixed methods retrospective cohort study to evaluate if a new program launched in 2018 involving a direct benefit transfer (DBT) improved the outcomes of patients with initial treatment of TB in the Bhavnagar district of Gujarat state in India,^4^ where the annual incidence of TB in 2020 represented a quarter of the world’s burden for that year, and is one of the most deprived populations among all low-income countries.

 The authors describe the synergy of the dual epidemics of TB and undernutrition and the country’s response to adapt the World Health Organization (WHO) guidelines on nutritional care and the DBT scheme for patients undergoing anti-TB treatment to meet their nutritional requirements.

 The quantitative component of the study evaluated if failure to receive the DBT was a predictor of unfavorable treatment outcomes, which was followed by a qualitative component of in-depth interviews among TB health visitors, treatment supervisors, the program coordinator and the district TB officer and patients with drug-sensitive pulmonary TB who had received the DBT. They found that among the 426 patients, only 37 had not received DBT and although 91% completed their treatment, non-receipt of DBT was associated with 5-fold odds (95% CI: 2-12) of unfavorable treatment outcomes on multivariable analysis. They concluded that DBT could improve treatment completion rates among patients with TB in their setting but, based on the interviews with TB officials and patients, all suggested the need to reach the poorest patients who did not own a bank account to receive the DBT and to increase the existing assistance under DBT with the provision of a monthly nutritious food-kit since the amount of the monthly DBT was not sufficient to buy food. Difficulties in the implementation process (lagged reception of installments, lack of a bank account) were issued by Dave and Rupani, as well as other authors. Other aspects of implementation and broadening of target population of CCT (ie, from rural to urban households) were also reported.^5^ Although DBT is intended to be used as nutrition support, a health visitor in Dave and Rupani’s paper reports money being spent with other purposes by some patients (“mostly spent on addiction”). Direct food supplementation was evaluated in some studies,^6,7^ with treatment default risk reduction effects of treatment ranging from 10% to 50%. Favorable results were also found when sputum smear or culture negative conversion rate – instead of TB treatment adherence – were evaluated as primary outcome.^8^ However, a Cochrane Systematic Review was unable to prove consistent improvement in TB outcomes.^9^

 Dave and Rupani’s study has some limitations. The authors state that it is one of the initial studies using a cohort design adjusting for important confounders, but only 37 (9%) of the participants did not receive the DBT and 91% completed treatment successfully, thus limiting the statistical power to evaluate the association of several variables with the outcome and control for several simultaneous potential confounders. Table 1 of Supplementary file 2 lists a number of variables failing to show statistical association with the primary outcome but showing wide confidence intervals, likely due to sample size constraints. The multiple logistic regression model shows the effect of not receiving DBT on the TB outcomes adjusting for 8 variables, which may contribute to the lack of precision of the effect estimates. Results are shown as odds ratio with 95% CI but do not include the actual numbers. The authors did not compare the baseline characteristics of the 2 groups which might have been helpful to identify those variables associated with not receiving the intervention and eventually obtain a propensity score to help adjust confounding effects in a subgroup in the context of a limited number of exposed individuals and those achieving the primary endpoint.

 Nevertheless, even with these limitations acknowledged by the authors, the study is a helpful contribution to the growing but yet insufficient evidence showing how social protection interventions improve treatment outcomes and play an important role to achieve TB control.

 There is some evidence that cash transfer interventions improve treatment outcomes in patients with active pulmonary TB in low- and middle-income countries, although the overall quality of this evidence is limited. A recently published systematic review and meta-analysis on cash interventions to improve TB outcomes^3^ reported final results on only 4 studies on specific and one study on a sensitive TB intervention. The authors found substantial heterogeneity in study designs but still concluded that cash transfer interventions for patients in low- and middle-income countries improved TB treatment outcomes. They also discussed that the evidence is still weak and only identified one randomized control trial of a social protection intervention integrating social support with CCTs in Peru.^10^

 Unfortunately, demonstrating that social protection directly improves a specific health outcome is not simple^11^ due to the difficulty to establish an undisputed causal relationship between social protection interventions and improvements of TB outcomes. Although there may be a strong conceptual framework linking upstream interventions to downstream outcomes, it may not be clear if social protection interventions improve clinical outcomes if confounding effects are not adequately accounted for.

 There are many methodological challenges to assess the effect and impact of policy interventions implemented in real life based on routinely collected data, incomplete registries or surveillance reports, all sources of observational data. Randomized trials are the gold standard to assess the effect of an intervention, but they can be logistically difficult, expensive, lengthy, and potentially unethical.^12^ In this context, and to assist a timely decision-making process, we need to obtain answers within the limits of other study designs and data sources conducting research that is both methodologically rigorous and politically relevant. It is true that assessing the effect of policies or interventions using observational data poses a challenge, which nevertheless can be reasonably overcome using adequate methods and acknowledging the potential limitations. In this scenario, observational and modeling studies shedding light on different strategies to achieve better treatment outcomes are welcome.

 Our group has worked with TB-affected households in the province of Buenos Aires, Argentina for the last 10 years. We conducted different studies evaluating strategies of system, social and economic support for patients with TB and also found that the CCTs may be a valuable health policy intervention to improve the control of TB in similar high-burden areas.^13^ Interestingly, just the registration for this financial incentive (ie, the *intent *to grant this CCT) and not the actual receipt of the cash had a significant effect on adherence to TB treatment, independently of modality of treatment received, age, educational and income level, employment and marital status, usual source of care and the availability of community programs, suggesting that part of the effect might be mediated by the patients’ perception of support from the health system. It was also found upon reanalysis (not published) that directed observed treatment scheme and baseline risk of treatment default acted as effect modifiers of CCT on TB outcomes. The results should encourage decision makers to facilitate and promote the implementation of these policies and increase the coverage to all TB patients and households living under vulnerable conditions.

 Conditional or unconditional cash transfer interventions have become some of the most popular forms of social protection approaches to improve clinical outcomes for TB.^14^ TB specific cash transfer interventions can contribute to offset costs caused by the disease, especially those related to travel to the clinic and buying food, but mainly by compensating indirect costs caused by loss of wages for those patients with precarious jobs, as it is the case for many. If they are conditional on the compliance with the treatment, it may also serve as an additional incentive to achieve the end of TB.

 But the concept of social protection involves a much wider range of policies and structural changes to help people move out of extreme poverty, and TB specific interventions are only limited to the duration of the treatment. Similarly to other conditions, TB is a disease tightly related to social and economic deprivation, and the direct and indirect costs associated with it can exacerbate poverty and increase the likelihood of adverse TB outcomes, very much like the circular cumulative causation mechanism proposed by Gunnar Myrdal in the theory of development economics.^15^

 It is paramount to better understand which forms of social protection interventions are most effective at improving outcomes for TB, the delivery methods and implementation strategies.

 Social protection is a human right and an essential component of any patient centred care strategy, especially in vulnerable groups struggling with TB and other poverty-related diseases. To expand the evidence base in addition to cost-mitigation strategies, rigorously designed modeling studies, cluster-randomized trials and pragmatic operational studies based on real world data are also needed to evaluate the impact of social protection interventions combining nutritional, psychosocial, and economic support with more upstream measures to reduce structural poverty such as education, housing and employment opportunity policies.

## Ethical issues

 Not applicable.

## Competing interests

 Authors declare that they have no competing interests.

## Authors’ contributions

 Both authors contributed equally to this work.
